# A versatile multimodal chromatography strategy to rapidly purify protein nanostructures assembled in cell lysates

**DOI:** 10.1186/s12951-023-01817-2

**Published:** 2023-02-24

**Authors:** Daniel L. Winter, Hélène Lebhar, Joshua B. McCluskey, Dominic J. Glover

**Affiliations:** 1 School of Biotechnology and Biomolecular Sciences, University of New South Wales, Sydney, Australia; 2 Recombinant Products Facility, Mark Wainwright Analytical Centre, University of New South Wales, Sydney, Australia

**Keywords:** Protein engineering, Protein–protein interactions, Nanostructures, Multimodal chromatography

## Abstract

**Background:**

Protein nanostructures produced through the self-assembly of individual subunits are attractive scaffolds to attach and position functional molecules for applications in biomaterials, metabolic engineering, tissue engineering, and a plethora of nanomaterials. However, the assembly of multicomponent protein nanomaterials is generally a laborious process that requires each protein component to be separately expressed and purified prior to assembly. Moreover, excess components not incorporated into the final assembly must be removed from the solution and thereby necessitate additional processing steps.

**Results:**

We developed an efficient approach to purify functionalized protein nanostructures directly from bacterial lysates through a type of multimodal chromatography (MMC) that combines size-exclusion, hydrophilic interaction, and ion exchange to separate recombinant protein assemblies from excess free subunits and bacterial proteins. We employed the ultrastable filamentous protein gamma-prefoldin as a material scaffold that can be functionalized with a variety of protein domains through SpyTag/SpyCatcher conjugation chemistry. The purification of recombinant gamma-prefoldin filaments from bacterial lysates using MMC was tested across a wide range of salt concentrations and pH, demonstrating that the MMC resin is robust, however the optimal choice of salt species, salt concentration, and pH is likely dependent on the protein nanostructure to be purified. In addition, we show that pre-processing of the samples with tangential flow filtration to remove nucleotides and metabolites improves resin capacity, and that post-processing with Triton X-114 phase partitioning is useful to remove lipids and any remaining lipid-associated protein. Subsequently, functionalized protein filaments were purified from bacterial lysates using MMC and shown to be free of unincorporated subunits. The assembly and purification of protein filaments with varying amounts of functionalization was confirmed using polyacrylamide gel electrophoresis, Förster resonance energy transfer, and transmission electron microscopy. Finally, we compared our MMC workflow to anion exchange chromatography with the purification of encapsulin nanocompartments containing a fluorescent protein as a cargo, demonstrating the versatility of the protocol and that the purity of the assembly is comparable to more traditional procedures.

**Conclusions:**

We envision that the use of MMC will increase the throughput of protein nanostructure prototyping as well as enable the upscaling of the bioproduction of protein nanodevices.

**Graphic Abstract:**

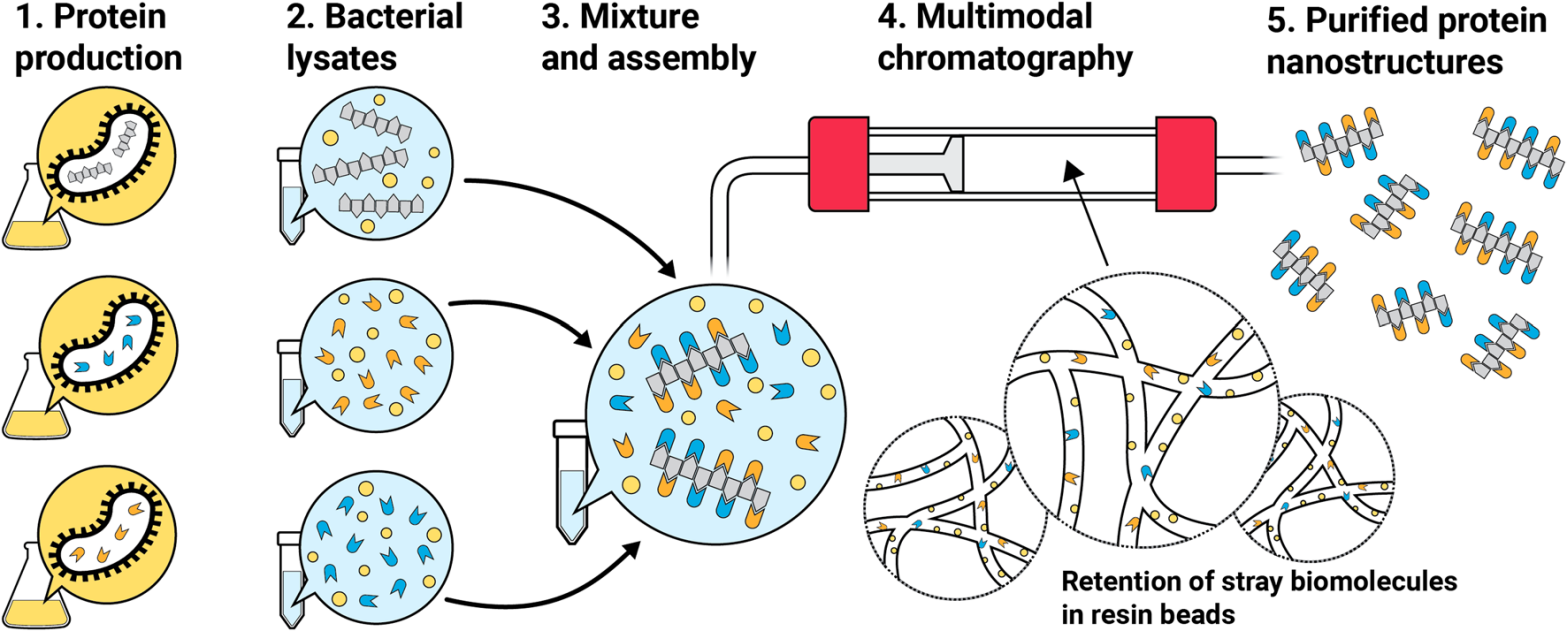

**Supplementary Information:**

The online version contains supplementary material available at 10.1186/s12951-023-01817-2.

## Background

The self-assembly of proteins can be harnessed to produce intricate nanostructures that can be functionalized with additional protein domains to create nanodevices for a variety of applications [1]. Functionalized protein nanostructures are generally composed of many different protein subunits, with some subunits assembling as an architectural scaffold [2–4], while other subunits serve as functional components to produce chemical activities. The functional protein subunits are attached to the scaffold, whereby their proximity and alignment results in emergent properties of the whole assembly. The alignment of functional domains on protein nanostructures include enzymes for multistep biocatalysis pathways [5–7], cell signaling domains for tissue engineering [8], and metalloproteins on filaments to produce electronically conductive nanowires [9], However, the fabrication of multicomponent protein complexes is laborious, as the different components must be recombinantly expressed and purified separately before assembly of the functional nanostructure can occur [10].

It would be advantageous to the field of nanobiotechnology if the fabrication steps required to produce a nanostructure could be reduced and the process standardized using scalable manufacturing methodology. In the case of a homomeric protein nanostructure such as a symmetrical cage, only one protein component needs to be expressed and assembled. In this simple scenario, protein production is typically performed by recombinant expression in a microbial host, whereupon the protein monomer self-assembles in vivo into a pre-defined structure [11]. However, more useful assemblies can be obtained by combining different protein components, including functional domains into structurally complex heteromeric nanostructures [1]. Our capacity to engineer intricate self-assembling protein complexes has dramatically increased with the design of sequence-specific binding domains [12–14], and orthogonal reactive interfaces that form covalent attachments between proteins [15, 16]. However, the production of heteromeric protein assemblies requires laborious procedures to separately express and purify each individual protein component, followed by nanostructure assembly and removal of unincorporated protein subunits. We thus sought to develop an assembly and purification strategy to purify heteromeric protein assemblies from bacterial lysates, which is also compatible with a broad range of pH and salt conditions and does not rely on the incorporation of chromatography affinity tags.

Capto Core 700 (CC700) is a multimodal chromatography (MMC) resin combining size-exclusion, ion exchange, and hydrophobic interaction chromatography to enable purification of large protein assemblies [17]. The resin is composed of beads whose surface is inactive and cannot bind to biomolecules. The pores of the CC700 beads have a large molecular weight cut-off (700 kDa), whereby assemblies larger than the pore size are excluded from entering the beads and are instead recovered in the chromatography flow-through. Smaller molecular assemblies, however, enter the pores and are retained inside the beads through interaction with the internal bead surface that has a mixture of hydrophilic and hydrophobic groups for molecule binding.

**Fig. 1:**
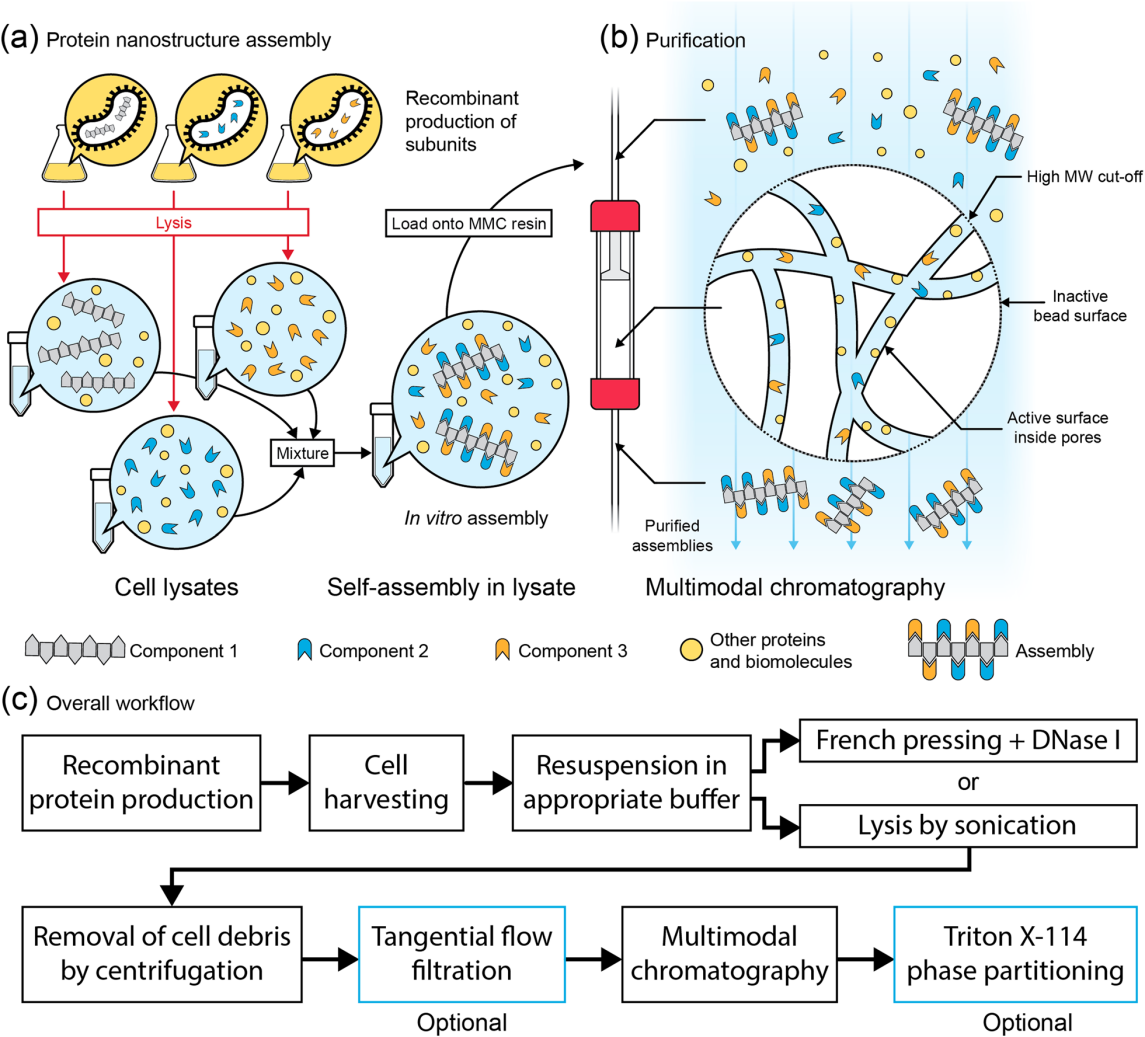
Recombinant production of protein nanostructures and their purification by multimodal chromatography. **a** The components of a protein assembly can be recombinantly produced in separate microbial strains. Following lysis of each bacterial culture, the lysates are mixed, wherein the components self-assemble into nanostructures. **b** Following assembly, the protein nanostructures can be purified from the rest of the lysate, including excess free components, using a multimodal chromatography (MMC) resin, Capto Core 700 (CC700). In CC700 MMC, only molecules that are smaller than the resin pores can enter the beads, where they bind to the active surface inside the beads. Because the assembled nanostructures are too large to enter the pores, and because the shell of the beads is inactive, the nanostructures do not bind to the resin and are recovered in the flow-through. **c** Diagram illustrating the purification procedure employed in this study. The TFF and TX-114 steps (blue squares) are optional and are useful to increase CC700 resin capacity and remove lipids and lipid-bound proteins, respectively

Previously, CC700 has been used to purify viruses from mammalian cell lysates [18], and recombinantly expressed viral-like particles [19, 20] and encapsulin nanocompartments [21]. However, we hypothesize that CC700 technology can be expanded to enable single-step assembly and purification of a functionalized heteromeric protein nanostructure. Here, we verified the robustness of our strategy by systematically comparing the purification yields of the protein gamma-prefoldin (γPFD), which self-assembles into filaments in the megadalton scale, at several salt concentrations and pH values. Subsequently, we demonstrated the filaments could be functionalized and purified directly from bacterial lysates. An engineered gamma-prefoldin fused to a SpyTag domain and three functional proteins fused to a SpyCatcher domain were recombinantly produced in separate *Escherichia coli* strains. Combining the four bacterial lysates triggered nanostructure assembly and functionalization (Fig. 1a) and enabled the full assembly to be purified using MMC, free of excess subunits that were not incorporated into the complex (Fig. 1b). To further improve purity, sample processing steps were tested both upstream and downstream of MMC, including tangential flow filtration (TFF) and Triton X-114 (TX-114) phase separation, which increased resin capacity or removed any remaining lipids and protein contaminants. The capacity to assemble complex assemblies and their subsequent purification using a rapid, standardized procedure will facilitate the production and upscaling of engineered protein nanostructures.

## Results and discussion

### CC700 enables robust purification of recombinant protein filaments from bacterial lysates

The combined assembly and purification of functional protein nanostructures from cell lysates will require solvent conditions that are compatible for each process. Thus, we performed a series of experiments to determine whether a commercially available MMC resin, Capto Core 700 (CC700), could efficiently purify protein nanostructures in varying pH and salt conditions and identify the optimal conditions for the purification of large protein assemblies. Specifically, we chose salt concentrations ranging from 0 to 1 M sodium chloride or 1 M ammonium sulfate, and pH values of either 6.5 or 8.0. The choice of salt, salt concentration, and pH should modulate the binding of proteins to the ion exchange and hydrophobic sites of CC700 resin [22] as well as the stability of large protein assemblies, and thereby affects the purity of the recovered protein nanostructures.

**Fig. 2:**
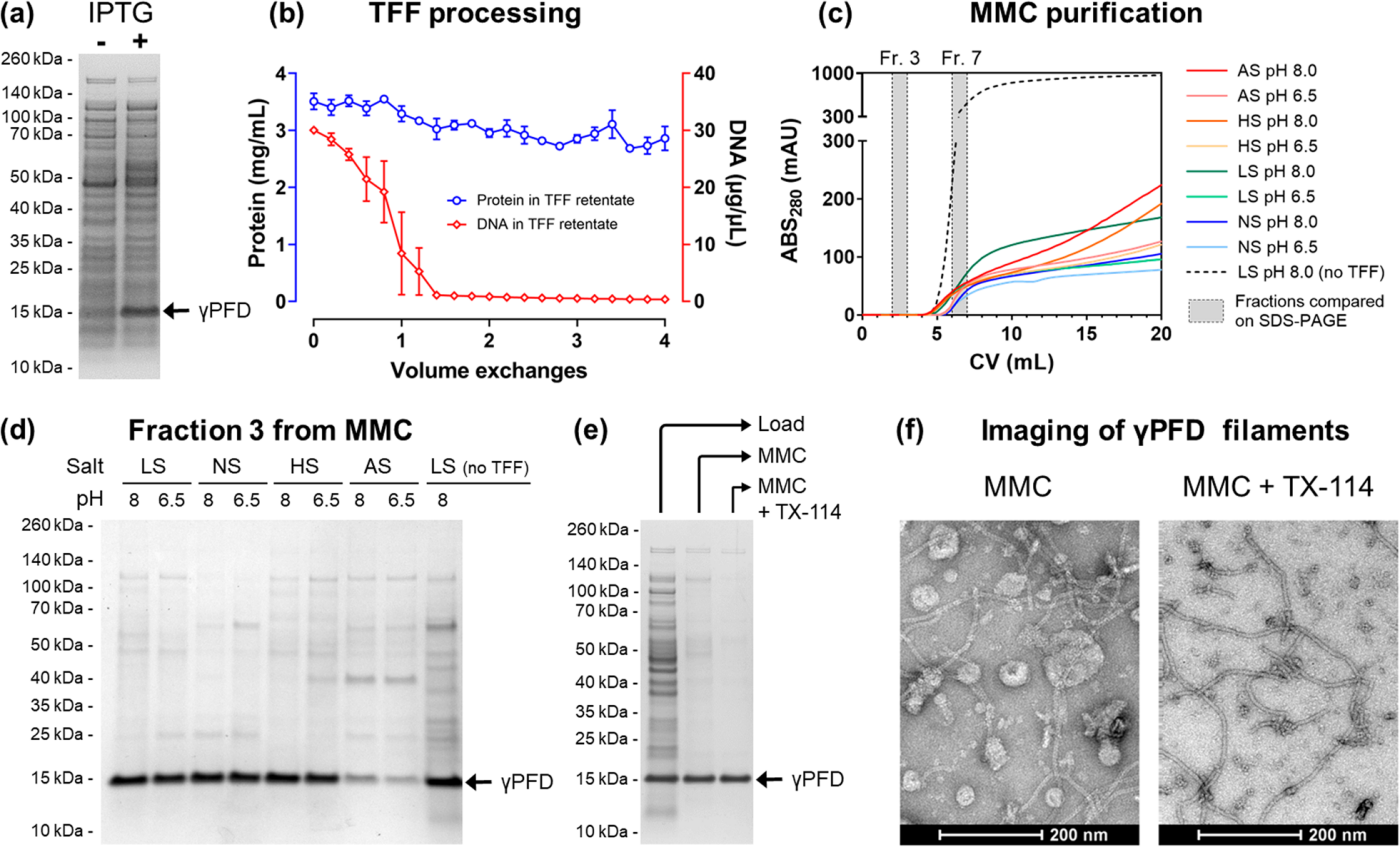
CC700 resin enables consistent protein assembly purification across a range of pH and salt conditions. **a** Recombinant expression of γPFD in *E. coli* following inducement with IPTG as shown by SDS-PAGE. **b** TFF with a 1 MDa filter does not remove contaminant proteins from a bacterial lysate but is effective in removing nucleic acids. **c** UV chromatograms of MMC purifications showing comparable performance in a range of buffered solvents. Pre-processing with TFF increases resin binding capacity. **d** Comparison of an early fraction from MMC performed with different buffered solvents confirms that MMC performs comparably in all solvents except for ammonium sulfate. Omitting TFF pre-processing results in lesser purity. **e** Large protein assemblies purified using MMC can be further purified with a TX-114 phase separation procedure. **f** Transmission electron microscopy reveals contamination, possibly lipidic in nature, without TX-114 post-processing. LS: low salt (100 mM NaCl); NS: no salt; HS: high salt (1 M NaCl); AS: ammonium sulfate (1 M); TX-114: Triton X-114

The archaeal molecular chaperone γPFD, which self-assembles into micrometer-long filaments and has previously been decorated with a variety of functional molecules [5, 9, 23, 24] was chosen as a protein nanostructure. Filaments of γPFD can be recombinantly produced (Fig. 2a) and are stable across a broad range of environmental conditions, which enabled us to compare the purification yield of the filaments using CC700 in the chosen range of salt and pH conditions. Filaments of γPFD were recombinantly expressed in *E. coli* and the cell pellets were lysed by French pressing in a low salt, phosphate-buffered solution. The resulting lysate was very viscous due to the presence of the large genomic DNA, and thus we treated the lysate with endonuclease DNase I to hydrolyze genomic and plasmid DNA into small oligonucleotides, removing all viscosity. Removal or destruction of genomic and plasmid DNA molecules is also important because such DNA polymers are large enough (several MDa) to be purified by CC700 and would result in DNA contamination of the purified protein assemblies.

An aliquot of the lysate was subjected to TFF with a large molecular weight cut-off (1,000 kDa) to compare the performance of TFF and CC700 for the removal of bacterial proteins from the lysate. TFF did not efficiently remove bacterial proteins from the γPFD-containing lysate, however nucleic acid content in the lysate was drastically reduced as measured by UV absorbance at 260 nm (Fig. 2b and Additional file 1: Fig. S1). Presumably, the oligonucleotides resulting from treatment of the lysate with DNase I passed through the TFF filter. The TFF-processed lysate was then divided and dialyzed into buffer solutions that varied in salt content and pH, totaling nine different loading conditions for MMC purification (Fig. 2c). The individual samples were loaded onto pre-packed 1 mL CC700 columns, with similar protein content and concentration (Additional file 1: Fig. S2). Chromatograms of MMC purifications indicated that protein content in the flow-through quickly increased after a few column volumes passed through the CC700 column, suggesting saturation of the resin, at which point the resin would cease to retain proteins smaller than the 700 kDa cut-off and the flow-through would not contain pure γPFD. We analyzed the early fractions from two MMC purification conditions (with and without TFF processing) by SDS-PAGE and confirmed that only the first few flow-through fractions contained purified γPFD and that TFF processing of the lysate improved purity (Additional file 1: Fig. S3). Two early fractions from each MMC purification condition were analyzed by SDS-PAGE to compare the purity of γPFD, which confirmed that the sample that was not subjected to TFF and thus contained higher nucleic acid concentration showed lesser purity compared to TFF-processed loads (Fig. 2d, Additional file 1: Figs. S3, S4). Presumably, oligonucleotides and other small biomolecules from the non-TFF-processed lysate were retained in the CC700 resin, leading to earlier saturation of the column binding capacity and cessation of effective purification. Of note, oligonucleotides bind to the core of the CC700 predominantly resin via charged interactions, which can be modulated by varying salt species, salt concentration and pH [25]. We only compared the effect of the removal of nucleic acids by TFF for one salt and pH condition, and it may be possible that other conditions would have exhibited better resin capacity without TFF pre-processing. However, given the very high concentration of DNA present in the bacterial lysates, it is unlikely that modulating charged interactions between nucleic acids and the CC700 resin alone would substantially improve the purification of protein assemblies.

Additional evidence of increased CC700 capacity following lysate processing by TFF are the elution chromatograms for each tested condition (Fig. 2c and Additional file 1: Fig. S5), which show a much steeper increase in UV absorbance at 280 nm across fractions for the sample that was not pre-processed using TFF compared to the TFF-processed conditions. Thus, TFF served to increase the binding capacity of CC700 resin for bacterial proteins by removing biomolecules (predominantly nucleic acids), which is useful for the scale up of MMC purifications. Altogether, most conditions performed very similarly in regard to column capacity to retain bacterial proteins, with very slight variation of the onset of resin saturation (Additional file 1: Fig. S5). One exception were MMC conditions that used 1 M ammonium sulfate instead of sodium chloride, which resulted in both bacterial proteins and γPFD being retained in the resin (Fig. 2d). Although the initial concentration of γPFD in the lysates buffered-exchanged into ammonium sulfate was identical to the other conditions (Additional file 1: Fig. S2), significantly less γPFD was recovered following MMC. Presumably, the high ionic strength of ammonium sulfate compared to sodium chloride is disrupting the association of γPFD monomers into filaments, resulting in retention of γPFD monomers or small oligomers in the resin and less recovery from the resin. This result highlights the importance of the choice of buffered solutions to retain fully assembled protein complexes during MMC purification.

Nonetheless, all purification conditions produced a trace amount of background protein contamination (Fig. 2d). We reasoned that protein contamination may result from proteins bound to bacterial lipid membrane fragments that are known to form vesicles cell lysates. The nonionic detergent TX-114 is commonly used to remove endotoxins (which are lipopolysaccharides that can aggregate into vesicles) from bacterial lysates and has been previously used as a downstream polishing step following MMC for the preparation of virus-like particles [19]. We thus attempted to improve the purity of γPFD following MMC by phase-separating lipids from proteins using TX-114 [26]. This procedure was shown to remove background contamination and improve the purity of γPFD (Fig. 2e). In addition, transmission electron microscopy (TEM) showed that non-protein particle contaminants, possibly lipidic in nature, were removed from MMC-purified γPFD following TX-114 phase separation (Fig. 2f). While the TX-114 phase separation was only performed on early MMC fractions which contained relatively pure γPFD, this simple polishing step could be used on additional fractions of interest to remove lipids and lipid-associated proteins, improving the overall yield of the complete procedure. Altogether, CC700 resin performed similarly well for most salt and pH conditions and should thus be compatible with most types of functionalized protein assemblies. Straightforward bacterial lysate pre- and post-processing using TFF and TX-114 phase separation, respectively, can be used to improve the capacity of MMC resin and the purification of large protein assemblies (Fig. 1c).

### Strategy to attach and interspace functional proteins to γPFD filaments

Single-step assembly and purification of functionalized protein nanostructures from bacterial lysates would be a useful methodology for the field of nanobiotechnology. Having verified the robustness of CC700 for the purification of filamentous γPFD, we examined if the filaments could be directly functionalized in bacterial lysates and purified as complete nanostructures using CC700 resin. Previously, γPFD has been decorated with varying amounts of the fluorescent proteins mCerulean3 (CFP) and mVenus (YFP) via SpyTag/SpyCatcher conjugation to demonstrate the controlled positioning of functional domains [5]. When aligned on γPFD filaments in nanometer-scale distances (< 10 nm), the energy from an excited CFP is transferred to YFP through Förster Resonance Energy Transfer (FRET), resulting in decreased CFP emission and increased YFP emission. Varying the ratio of the two fluorescent proteins relative to the γPFD subunits enabled a controlled distribution and spacing of the fluorescent proteins along the filament. In this approach, three fusion proteins are recombinantly expressed and individually purified, including: γPFD C-terminally fused to a SpyTag domain (γPFD-SpyT), CFP C-terminally fused to a SpyCatcher domain (CFP-SpyC), and YFP C-terminally fused to a SpyCatcher domain (YFP-SpyC). Functionalized filaments were created by first assembling γPFD-SpyT into filaments, before mixing with CFP-SpyC and YFP-SpyC that conjugate to the SpyTags protruding from the γPFD-SpyT domains. However, this approach to build functionalized nanostructures was laborious and required each protein subunit to be separately purified and protein concentration determined to ensure appropriate molar ratios of the different protein subunits are combined. Furthermore, excess CFP-SpyC or YFP-SpyC that was not conjugated to γPFD-SpyT or excess γPFD-SpyT that was not functionalized was present in some mixtures, which requires additional purification steps for their removal. Of note, SpyTag/SpyCatcher conjugation is preferred to the direct genetic fusion of γPFD to other proteins for several reasons: (i) γPFD-SpyT readily forms filaments, whereas fusing γPFD to larger proteins can impair filament formation due to steric hinderances; (ii) in the cases where a γPFD fusion retains its filamentous structure, the resulting homomeric filament is only decorated with a single type of protein; to obtain a heteromeric filament requires the denaturing the different filaments, mixing the different filament subunits, followed by refolding to incorporate the different γPFD subunits into single filaments [23]. In contrast, SpyTag/SpyCatcher conjugation enables the concurrent functionalization of γPFD-SpyT with different proteins.

**Fig. 3:**
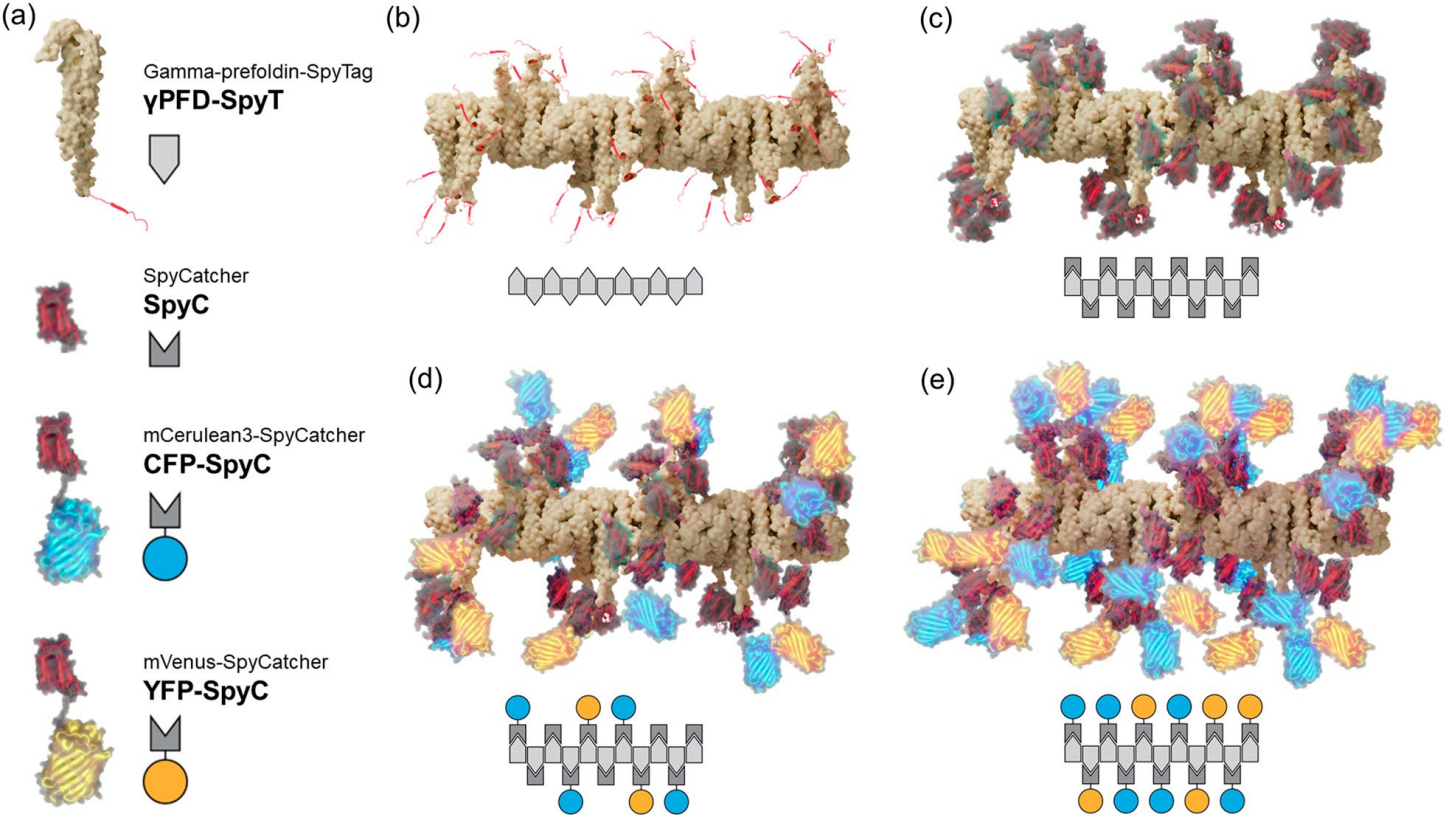
A strategy to attach and interspace functional proteins to γPFD filaments. **a** Predicted structural protein models of γPFD-SpyT and the functionalization subunits that are also represented as schematic icons. **b** Structural model of a γPFD-SpyT filament with SpyTag domains protruding from the C-termini of each subunit. **c** The SpyTag domains on γPFD-SpyT filaments can be blocked by the covalent attachment of SpyC subunits. **d** Attachment of CFP-SpyC and YFP-SpyC to the γPFD-SpyT filament via SpyTag/SpyCatcher covalent interactions. In this model, the CFP-SpyC and YFP-SpyC are randomly interspaced by SpyC. **e** Maximal density of CFP-SpyC and YFP-SpyC subunits attached to a γPFD-SpyT filament should be achieved by omitting the SpyC subunit

**Fig. 4:**
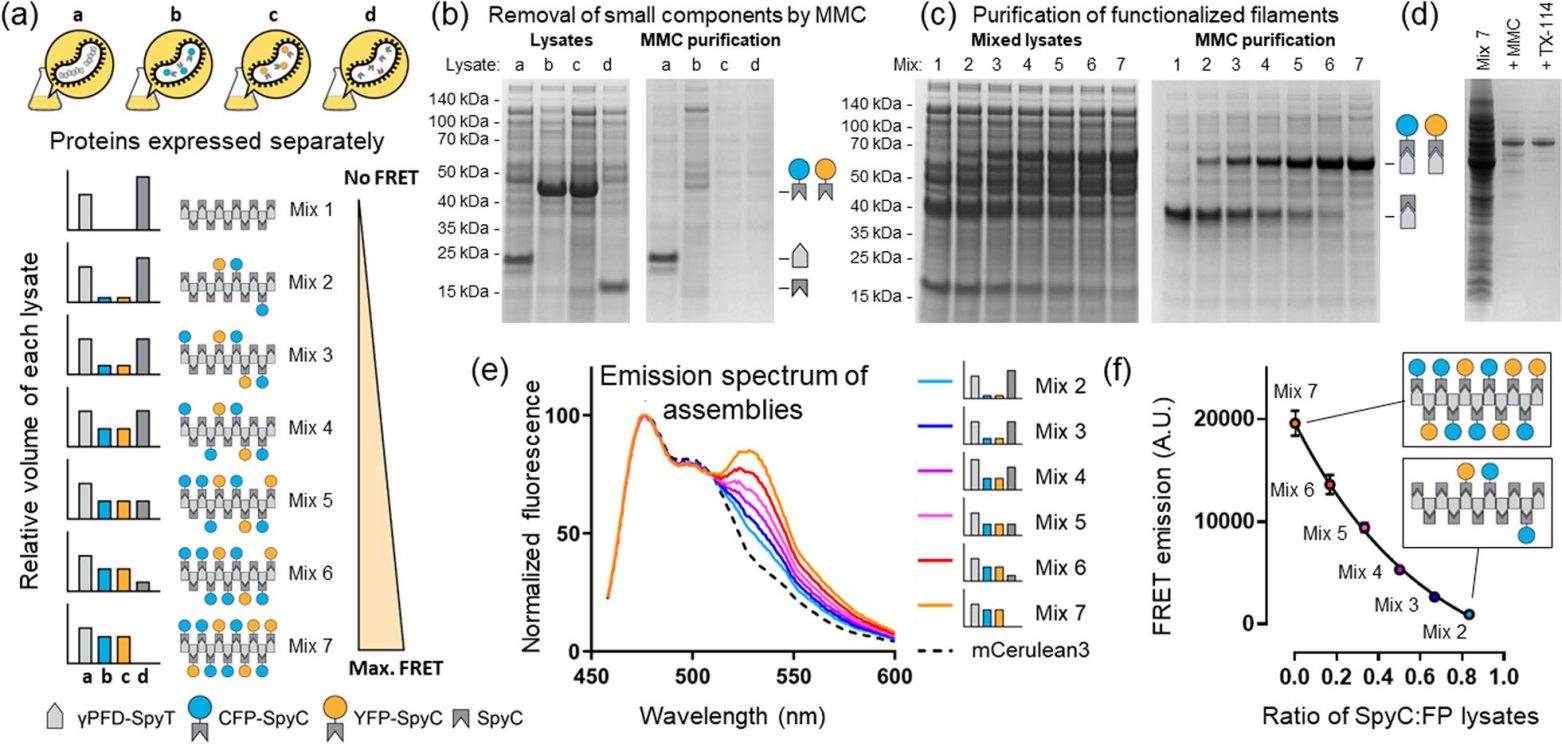
In vitro assembly and MMC purification of a functionalized γPFD protein filament. **a** Strategy for the assembly of functionalized protein filaments by combining bacterial lysates containing different protein components. The bars indicate the relative volumes of lysates a, b c, and d (which correspond to γPFD-SpyT, CFP-SpyC, YFP-SpyC, and SpyC, respectively) used in the different mixtures. Increasing the ratio of SpyC in the lysate mixture interspaces the fluorescent proteins CFP-SpyC and YFP-SpyC attached to γPFD-SpyT. **b** SDS-PAGE showing recombinant protein in bacterial lysates and after MMC. The large γPFD-SpyT filaments (lane a) pass through the resin whereas the small protein components are retained in the resin. **c** Lysate mixtures before and after MMC. Lysate mixtures demonstrate the conjugation of varying ratios of SpyC and the fluorescent proteins CFP-SpyC and YFP-SpyC to filaments and subsequent purification using MMC. **d** Improved purification of functionalized filaments using a larger volume of CC700 resin followed by TX-114 phase separation. **e** Fluorescence emission spectra of each functionalized filament preparation. Decreasing the amount of SpyC increases the likelihood that CFP-SpyC and YFP-SpyC are located close to each other on the γPFD-SpyT filament, increasing FRET (indicated by the higher emission at 527 nm relative to 476 nm). **f** The FRET emission of each filament preparation exhibits a strong correlation with the amount of SpyT used as a spacer between the fluorescent proteins. Data points represent the average of two experiments

In contrast to the previously published method, using CC700 should enable the purification of the functionalized filaments and the removal of unincorporated CFP and YFP subunits in the same step. We therefore adapted the SpyTag/SpyCatcher conjugation strategy for the functionalization of γPFD in bacterial lysates. In addition to the γPFD-SpyT, CFP-SpyC, and YFP-SpyC fusion proteins, a fourth protein will be recombinantly produced, the SpyCatcher domain (SpyC) that will be used to control the spacing of fluorescent subunits on the filament (Fig. 3). In this new approach, filament functionalization will be achieved by mixing bacterial lysates that contain recombinant γPFD-SpyT filaments with lysates each containing SpyC, CFP-SpyC, and YFP-SpyC, followed by purification of functionalized filaments by MMC. Varying the relative amounts of each lysate should modulate the average distance between the two fluorescent proteins aligned on filaments, and thereby modulate the FRET signal produced by functionalized filaments (Fig. 4a). Control over the average distance will be achieved by using the SpyC component to block SpyTags protruding from the filamentous γPFD-SpyT and thereby render these domains unavailable to bind the SpyCatcher domains of either CFP-SpyC or YFP-SpyC (Fig. 3). Therefore, the amount of FRET will be inversely proportional to the amount of SpyC lysate added to the mixture of γPFD-SpyT, CFP-SpyC, and YFP-SpyC lysates. The use of the additional SpyC component to block SpyTags has the additional advantage of enabling assembly conditions to contain excess concentrations of CFP-SpyC and YFP-SpyC to ensure near complete γPFD functionalization.

### MMC purification of a functionalized protein filament assembled in vitro

DNA plasmids were prepared encoding the recombinant expression of the four recombinant proteins γPFD-SpyT, CFP-SpyC, YFP-SpyC, and SpyC. The successful expression of the four proteins in *E. coli* was confirmed by SDS-PAGE of the lysates (Fig. 4b). Lysates were prepared by sonication of resuspended cells in a low salt, phosphate-buffered solution; as sonication shears genomic and plasmid DNA, the resulting lysate was not viscous, and DNase I treatment was omitted. Each lysate was subjected to MMC which showed that, as expected, the megadalton γPFD-SpyT filaments could be purified using CC700 resin. However, the CFP-SpyC, YFP-SpyC, and SpyC proteins were retained in the CC700 resin (Fig. 4b). These results confirmed that in addition to removing bacterial proteins, CC700 can also be used to remove unincorporated small protein components of a protein nanostructure.

Next, γPFD filaments were functionalized with fluorescent proteins by mixing the four different bacterial lysates in varying volumetric ratios to demonstrate control over the spacing between functional domains. For these mixtures, the volume of lysate containing γPFD-SpyT was kept constant and the volumes of lysates containing CFP-SpyC, YFP-SpyC, and SpyC were varied. From a qualitative analysis of the SDS-PAGE results (Fig. 4b), we observed that the CFP-SpyC, YFP-SpyC, and SpyC functional components were expressed in equal or higher amounts than γPFD-SpyT. Lysate volumes were chosen that ensured an excess of the functional components relative to γPFD-SpyT. Specifically, each mixture contained a total volume of 5 mL, of which 2 mL corresponded to the lysate containing γPFD-SpyT and the remaining 3 mL corresponded to various ratios of the lysates containing CFP-SpyC, YFP-SpyC, and SpyC (Fig. 4a).

Following the mixing and incubation of lysates, conjugation between γPFD-SpyT and the other SpyCatcher-containing components was verified by SDS-PAGE (Fig. 4c). An SDS-PAGE gel showed the formation of higher molecular weight species corresponding to a covalent attachment between γPFD-SpyT subunits of filaments and SpyC, CFP-SpyC or YFP-SpyC to form, respectively, γPFD-SpyT/C, γPFD-Spy-CFP and γPFD-Spy-YFP. Importantly, bands corresponding to excess SpyC, CFP-SpyC and YFP-SpyC were observed by SDS-PAGE, which are components not attached to γPFD-SpyT and therefore not incorporated into the protein filaments. Moreover, the band corresponding to γPFD-SpyT was not observed in the lysate mixtures, confirming that γPFD-SpyT subunits were completed conjugated with either of the three other subunits, and therefore were the limiting component in the functionalization reactions.

The presence of unincorporated components in the lysates enables evaluation of the ability of CC700 to remove unincorporated components of a protein nanostructure. The lysates mixtures containing the protein assemblies were subjected to MMC purification (Fig. 4c), this time manually using loose CC700 resin in gravity columns instead of using an automated FPLC system. The γPFD-SpyT/C, γPFD-Spy-CFP and γPFD-Spy-YFP subunits from the different assemblies were able to be isolated by MMC, whereas the excess SpyC, CFP-SpyC and YFP-SpyC were almost completely retained in the MMC beads. The MMC purification results suggested that functionalized filaments were created upon mixing of the lysates, that the filaments were composed of fluorescent (γPFD-Spy-CFP and γPFD-Spy-YFP) and non-fluorescent (γPFD-SpyT/C) conjugated subunits, and that the composition of the functionalized filaments were proportionate to the volumetric ratios of the different lysates used for functionalization.

The manual MMC purification of functionalized filaments (Fig. 4c), without prior TFF processing and without the UV monitoring and automated fractionation afforded by an FPLC system resulted in some background contamination. A second round of MMC demonstrated the removal of these bacterial protein contaminates, and the γPFD-SpyT functionalized with only CFP-SpyC and YFP-SpyC was further purified by TX-114 phase separation to remove lipidic vesicles from the MMC flow through (Fig. 4d). The morphology of the CFP-SpyC and YFP-SpyC functionalized γPFD-SpyT filaments was examined using by TEM (Additional file 1: Fig. S6). Filaments of γPFD-SpyT that were functionalized with the fluorescent proteins differed in morphology to non-functionalized filaments, which resembled concatenated “beads”. A FRET assay was used to confirm co-purification of the fluorescent proteins and non-fluorescent SpyCatcher protein with the filaments was due to their incorporation into filament nanostructures. FRET should occur when the mCerulean3 of γPFD-Spy-CFP subunits is less than 10 nm from the mVenus of γPFD-Spy-YFP. The likelihood of the donor and acceptor fluorescent subunits aligned within 10 nm along the filament scaffold should decrease as the number of the non-fluorescent γPFD-SpyT/C subunits increase, and the fluorescent subunits become progressively interspaced. Therefore, the lysate mixtures containing more CFP-SpyC and YFP-SpyC relative to SpyC should result in filaments that produce more FRET signal (Fig. 4a). A 433 nm light source was used to excite CFP and the emission spectrum was recorded for each assembly (Fig. 4e). The emission at 533 nm (optimal emission of YFP) relative to emission at 476 nm (optimal emission of CFP) increased with a composition higher in fluorescent subunits, which is indicative of increasing FRET. The deconvoluted FRET signal for each assembly is shown in Fig. 4f and illustrates the steady decrease in FRET signal as the fluorescent subunits become increasingly interspaced by γPFD-SpyT/C subunits.

In summary, the composition of the filaments could be controlled by combining varying volumes of bacterial lysates that contain functional domains and the resulting functionalized filaments purified in a single step using CC700. We chose to work with CFP-SpyC, YFP-SpyC, and SpyC as model functional proteins due to the convenience of FRET assays to investigate filament composition, but γPFD-SpyT could be functionalized with any combination of practically significant proteins, including enzyme cascades [5], and purified in the same manner. Using standard procedures, the functionalized γPFD assemblies would have required four separate purifications (and potentially buffer-exchanging steps) to produce the nanostructure components, followed by additional polishing steps to remove excess free components after assembly. Using CC700, no purification of the individual components was required, and purification of the large assembly was achieved concomitantly to the removal of unincorporated subunits in a single step. In our experiments, a second round of MMC was performed to improve purification, however the same result could be obtained through the use of a CC700 column with a greater volume of resin and binding capacity to increase the purity of nanostructures in eluted early fractions. Thus, the production of protein nanostructures can be greatly accelerated by use of CC700 resin as the method of purification.

### In vivo assembly of a loaded protein cage and its purification by MMC

**Fig. 5:**
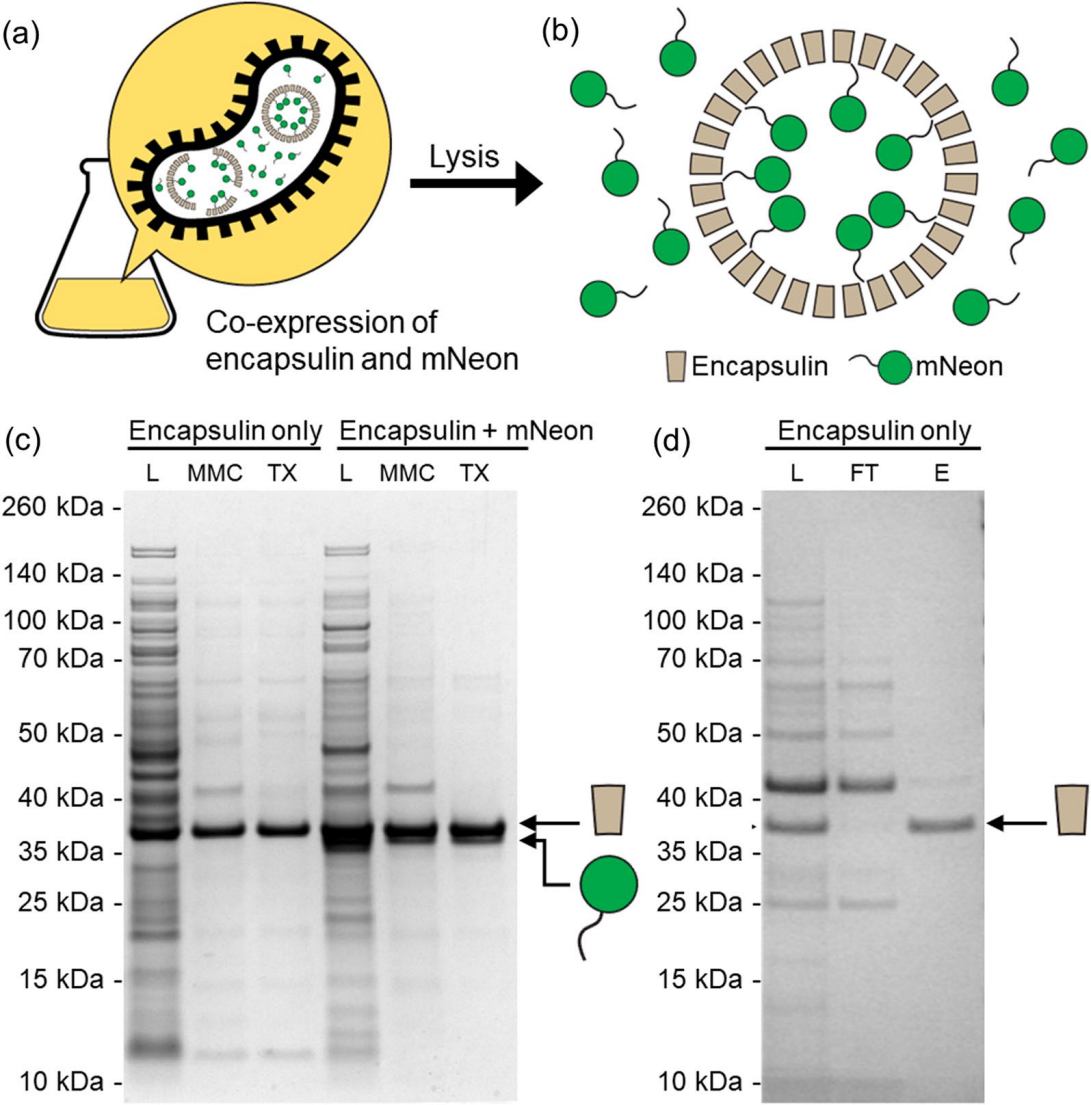
Purification of a loaded protein cage assembled within *E. coli* cells via co-expression. **a** Encapsulin and mNeon are co-expressed in *E. coli* and a fraction of mNeonGreen is encapsulated upon interaction with encapsulin via a target peptide and self-assembly of encapsulin into a protein cage. **b** Upon cell lysis, the lysate contains encapsulin cages loaded with mNeon and excess mNeon outside the cage. **c** SDS-PAGE showing the successful purification of encapsulin (non-loaded) and encapsulin loaded with mNeon via MMC followed by TX-114 phase partitioning. **d** SDS-PAGE showing the successful purification of encapsulin (non-loaded) using anion exchange membrane adsorbers. L: load; MMC: multimodal chromatography; TX: Triton X-114 phase partitioning; FT: flow-through; E: eluate

The versatility of MMC purification of multicomponent protein nanostructures was further demonstrated through the purification of a loaded protein cage that was assembled within *E. coli* cells. The self-assembling nanocompartment was an encapsulin from *Quasibacillus thermotolerans*, which when fully assembled, consists of cage structure of 240 encapsulin subunits, each with molecular weight of 32.2 kDa, for a total of 7.7 MDa and a diameter of 42 nm [27]. The *Q. thermotolerans* encapsulin was recombinantly co-expressed with the fluorescent protein mNeonGreen fused to a short targeting peptide (hereafter, mNeon) that promotes its association with the internal side of encapsulin, effectively packaging mNeon inside the protein cage (Fig. 5a). This strategy has been applied to load diverse proteins inside encapsulin cages [28, 29] and shows potential for the creation of drug delivery systems [30], bioreaction nanocompartments [29, 31], and vaccines [21].

Co-expression of encapsulin and mNeon in a single *E. coli* strain produced a fully assembled and loaded protein cage in vivo, which avoids the need to mix lysates containing different protein components for nanostructure assembly. As the 240-subunit encapsulin can interact, on average, with 42 cargo proteins [27], excess mNeon will be found outside the encapsulin cage if the two proteins are co-expressed at similar levels, and must be removed alongside bacterial proteins (Fig. 5b). Bacterial lysates containing encapsulin (non-loaded) and encapsulin loaded with mNeon were subjected to the established MMC nanostructure purification protocol. In both cases, the large encapsulin cages were completely recovered using MMC, with subsequent TX-114 phase partitioning shown to further improve purity (Fig. 5c). For the co-expression of encapsulin with its mNeon cargo, a high amount of both proteins was observed in the bacterial lysate (Fig. 5c); although the two proteins have a similar molecular weight, two overexpression bands can be distinguished by SDS-PAGE. Following MMC, encapsulin was fully recovered, but the band corresponding to mNeon showed a marked decrease in intensity, demonstrating the removal of excess mNeon that was not packed inside the encapsulin cages. TEM imaging of the samples confirmed the successful assembly of the encapsulin nanocompartments (Additional file 1: Fig. S6).

Finally, we sought to compare our MMC nanostructure purification strategy with anion exchange (AEX), which is a more traditional approach for protein purification. We performed the purification of non-functionalized γPFD and a non-loaded encapsulin from bacterial lysates using a commercial AEX membrane adsorber with quaternary ammonium groups (Sartobind Q Nano). Both γPFD and encapsulin could be purified using a sodium chloride gradient (Fig. 5d and Additional file 1: Fig. S7). Comparing the SDS-PAGE results in Figs. 5 and Additional file 1: Fig. S7, AEX showed better purity results. However, in these experiments, no excess functional protein subunits were present in the bacterial lysate. Thus, the capacity of membrane adsorbers to remove unincorporated subunits and the effect of the presence of additional recombinant proteins on the capacity of the adsorber could not be evaluated. Importantly, DNA and metabolites would compete with proteins for binding to the ammonium groups during AEX, and removal of these molecules (for example, using TFF) would also be necessary to optimize the use of high flow membrane adsorbers for the purification of protein nanostructures. Another disadvantage of AEX compared to MMC is the requirement for buffer exchanging after purification, whereas MMC can be performed directly in the final desired buffered solution.

## Conclusions

Herein, we have demonstrated a simple MMC method using CC700 resin to purify recombinantly produced protein nanostructures. Our method bypasses the need to separately purify each protein component, works in a wide range of biocompatible solvents, and removes both bacterial proteins and excess recombinant proteins that do not integrate the nanostructure in a single step. In the present study, these experiments have been performed at a small scale, however the method is inherently scalable. Furthermore, we presented both pre-processing (TFF) and post-processing (TX-114 phase separation, AEX with high flow membrane adsorbers) methods that can be used to improve the quality of the purification. Many more downstream polishing procedures exist [32] that should be compatible with CC700, especially in the context of large-scale purifications.

Taking together all of our observations, we propose two main areas of application of MMC for the purification of heteromeric protein assemblies. First, MMC is useful to simplify and downscale purifications for the rapid prototyping of different nanostructures that vary in the identities or ratios of subunits. The nanostructures can be assembled by mixing different bacterial lysates at small scale, or following co-expression of different subunits in vivo, then subjected to MMC to remove unincorporated subunits and the majority of bacterial protein contaminants in a single step. The prototyping of designs generally does not require highly pure samples, but higher purity can be achieved by increasing the CC700 resin volume or employing TX-114 phase partitioning as a polishing step. Second, MMC can be used for the upscaling of purifications. However, the large-scale production of protein nanostructures for practical applications requires high purities to be achieved. In this context, MMC should be combined with more stringent polishing steps, such as ion exchange technologies amenable to the retention of large protein structures, exemplified here with the use of AEX membrane adsorbers. In both scenarios (small-scale prototyping and large-scale production), the procedure can be automated to perform as many cycles of purification and clean-up as necessary to accelerate the speed and improve the final yield. Importantly, the yield of nanostructures purified using CC700 is related to the resin’s capacity to retain proteins smaller than 700 kDa. In our study, we calculated that the protein content of approximately one gram of wet bacterial cells can be removed using 10 mL of CC700 resin; the capacity of CC700 could be improved almost two-fold upon TFF-processing to remove nucleic acids and metabolites. To improve the yield of the protein assembly itself requires the optimization of the heterologous recombinant production of each protein subunit.

We also envision that with careful genetic encoding of the protein components, it may be possible to fully assemble heteromeric protein nanostructures of greater complexity in vivo via the co-expression of the protein components, followed by purification of the nanostructures by MMC. Such a strategy could further reduce the time and costs to produce protein nanodevices. Orthogonal protein interfaces and Tag/Catcher domains have been shown to guide the association of proteins in vivo [14, 33–36], opening a path for the in vivo preparation of protein nanostructures and nanodevices. A major challenge will be the control of the total and relative expression levels of each protein component to ensure the correct self-assembly and minimize the excess of free protein components that do not partake in the assembly. Novel strategies for the regulation of protein co-expression [37, 38] will need to be explored to achieve this vision.

## Methods

### Preparation of bacterial expression strains

Plasmids for bacterial expression of wild type γPFD, γPFD-SpyTag-6 × His, SpyCacther-6 × His, mCerulean3-SpyCatcher-6 × His, and mVenus-SpyCatcher-6 × His were prepared using Gibson assembly with pET-19b (Novagen) as the plasmid vector. A linear pET-19b backbone was produced by polymerase chain reaction (PCR), and the insert DNA fragments were prepared by either PCR or commercially synthesized by Integrated DNA Technologies. Plasmids encoding the expression of *Q. thermotolerans* encapsulin and the co-expression of encapsulin and mNeonGreen were a gift from Dr Yu Heng Lau (University of Sydney). Assembled DNA plasmids were transferred into competent T7 Express *E. coli* cells (New England Biolabs) and colonies selected on lysogeny broth (LB) agar plates supplemented with 50 µg/mL of ampicillin. The fidelities of expression plasmids were verified by Sanger sequencing at the Ramaciotti Centre for Genomics at the University of New South Wales.

### Expression of protein components and preparation of lysates

Overnight cultures were prepared for each strain by inoculating 5 mL LB medium supplemented with 50 µg/mL of ampicillin with a single colony. The next day, 250 mL baffled flasks containing 100 mL of LB medium with ampicillin were inoculated with 1 mL of an overnight culture and grown at 37 °C with shaking (200 RPM) until an optical density of 0.6 at A_600_. Subsequently, protein expression was induced for 15 h at 20 °C with the addition of isopropyl β-d-1-thiogalactopyranoside (IPTG) to a final concentration of 1 mM. Cells were harvested by centrifugation then resuspended in phosphate-buffered saline (50 mM sodium phosphate, 100 mM sodium chloride, pH 8.0) using 10 mL of buffer per gram of wet cell weight. Cell lysis was performed by sonication in. Cell debris were removed by centrifugation at 20,000*g* followed by filtration using 0.22 µm Millex syringe filters (Merck). Cleared lysates were used for subsequent protein assembly experiments. For the co-expression of encapsulin and mNeonGreen, the same procedure was followed, except that the LB medium was complemented with both 50 µg/mL of ampicillin and 25 µg/mL of kanamycin.

A similar procedure was performed for large-scale expression of wild type γPFD, except that fifteen 2.5 L baffled flasks, each containing 1 L of LB medium with ampicillin were inoculated. Inducement of protein expression was performed at an optical density of 0.6 at A_600_ followed by 15 h incubation at 20 °C with shaking (200 RPM). The cells were harvested by centrifugation and resuspended in 2 L phosphate-buffered saline (50 mM sodium phosphate, 100 mM sodium chloride, pH 8.0). Cell lysis was performed by French pressing at 15,000 psi. Bovine deoxyribonuclease I (Sigma-Aldrich) was added to a final concentration of 0.1 mg/mL to degrade genomic and plasmid DNA and reduce the viscosity of the lysate. The lysate was clarified by centrifugation at 10,000*g* and subjected to tangential flow filtration (TFF) as described below. The TFF-processed lysate was split into volumes of 150 mL, which were either stored at 4 °C or buffer-exchanged using SnakeSkin dialysis tubing (Thermo Fisher) with a 10 kDa cut-off prior to storage. The buffers used for buffer exchange are indicated in Table 1.

**Table 1:** Buffer solvents used for CC700 purification

Buffer name	Composition	Usage
No salt/low pH	50 mM sodium phosphate; pH 6.5	Buffer exchange
No salt/high pH	50 mM sodium phosphate; pH 8.0	Buffer exchange
Low salt/low pH	50 mM sodium phosphate; 0.1 M sodium chloride; pH 6.5	Buffer exchange
Low salt/high pH	50 mM sodium phosphate; 0.1 M sodium chloride; pH 8.0	Lysis of cells
High salt/low pH	50 mM sodium phosphate; 1 M sodium chloride; pH 6.5	Buffer exchange
High salt/high pH	50 mM sodium phosphate; 1 M sodium chloride; pH 8.0	Buffer exchange
Ammonium sulfate/low pH	50 mM sodium phosphate; 1 M ammonium sulfate; pH 6.5	Buffer exchange
Ammonium sulfate/high pH	50 mM sodium phosphate; 1 M ammonium sulfate; pH 8.0	Buffer exchange

### Tangential flow filtration

Tangential flow filtration was performed using a Labscale TFF System (Merck) and a Pellicon XL50 with Ultracel 1000 kDa membrane (Merck) using a feed flow rate of 20 mL/min and a transmembrane pressure maintained at 3–5 psi. A constant volume diafiltration was performed, where the retentate volume was held constant at 250 mL and the diafiltration buffer was added to the retentate at the same flow rate as the permeate. The experiment was stopped once 4 volumes of buffer were diafiltered with the total permeate volume reaching 1 L. Sampling of retentate and permeate was undertaken every 50 mL of permeate produced using sampling valves located on the retentate and permeate port of the diafiltration cassette. DNA content and protein content were quantified in each sample by UV absorbance at 260 nm using a NanoDrop One device (Thermo Fisher) and by using the Pierce BCA Protein Assay Kit (Thermo Fisher), respectively.

### Purification by multimodal chromatography

For the testing of different solutions, MMC was performed with 1 mL HiTrap columns loaded with Capto Core 700 (Cytiva) and an ÄKTA pure 25 FPLC system (Cytiva) to automate the process. Each column was equilibrated with 10 column volumes (CV) of one of eight buffered solutions (see Table 1) followed by the application of 20 CV of bacterial lysate (previously dialyzed in the same solvent, see above) containing recombinant γPFD. The flow-through was collected in 1 mL volume fractions. After each run, the columns were cleaned with 10 CV of distilled water, 10 CV of a cleaning-in-place solvent (1 M sodium hydroxide in 30% (v/v) isopropanol), 10 CV of distilled water, and re-equilibrated with one of the eight buffers being tested (Table 1). All steps were performed at 0.5 mL/min flow rate. In all other experiments, MMC was performed by gravity flow using Poly-Prep Chromatography Columns (Bio-Rad) loaded with up to 2 mL of Capto Core 700 resin. The procedure was essentially the same, except all steps were performed manually.

### Triton X-114 phase partitioning

We followed the method described in [39], with slight modifications, to remove lipids and lipid-associated proteins from selected MMC fractions. Briefly, 5 μL of TX-114 were added to 500 μL of purified protein assemblies, followed by vigorous vortexing, resulting in a cloudy mixture. The sample was left on ice for 10 min, at which point the solution became clear, indicating complete miscibility of water with TX-114. The sample was vortexed once more, then incubated for 5 min at 37 °C in a heating block, resulting in cloudy mixture indicating phase separation of TX-114. The sample was centrifuged for 1 min at 13,000*g* in a benchtop centrifuge. After centrifugation, two phases could be observed, with the lower phase (approximately 50 μL) corresponding to the detergent phase. The upper phase was carefully transferred to a clean tube for subsequent analyses.

### Assembly of functionalized γPFD filaments

Cleared lysates prepared by sonication containing recombinant γPFD-SpyT, CFP-SpyC, YFP-SpyC, and SpyC were mixed in various ratios in a total volume of 5 mL (Table 2) in 15 mL tubes. Lysates containing CFP-SpyC, YFP-SpyC, and SpyC were mixed first, and only then the lysate containing γPFD-SpyT scaffold was added, to ensure SpyTag/SpyCatcher conjugation started at the same time for all three functional proteins. The mixtures were gently agitated on a rotary tube mixer for 1 h at room temperature to allow the completion of the Tag/Catcher conjugation.

**Table 2:** Volumes of mixed lysates used for each functionalized filament

Lysate	Mix 1 (mL)	Mix 2 (mL)	Mix 3 (mL)	Mix 4 (mL)	Mix 5 (mL)	Mix 6 (mL)	Mix 7 (mL)
γPFD-SpyT	2	2	2	2	2	2	2
CFP-SpyC	0	0.25	0.5	0.75	1	1.25	1.5
YFP-SpyC	0	0.25	0.5	0.75	1	1.25	1.5
SpyC	3	2.5	2	1.5	1	0.5	0

### Fluorescence measurements

Fluorescence and Förster resonance energy transfer (FRET) measurements were performed with a CLARIOstar plate reader (BMG Labtech). For the measurement of fluorescence emission spectra, the excitation wavelength was set 433 nm (10 nm width) and the fluorescence emission was recorded from 455 to 600 nm wavelengths (10 nm width). For the measurement of FRET emission, three fluorescence values were recorded, with the following excitation/emission wavelengths: 433/476 nm (mCerulean3), 515/527 nm (mVenus), and 433/527 nm (FRET). The method described by Song et al. [40] was used to subtract background fluorescence resulting from the direct activation of mCerulean3 and mVenus and obtain the value of the FRET emission.

### Anion exchange with membrane adsorbers

Membrane chromatography was performed on a Sartobind Q Nano 1 mL, 4 mm bed height (Sartorius) using an ÄKTA Pure FLPC system (Cytiva). The membrane was equilibrated with 10 CV of no salt buffer (50 mM sodium phosphate; pH 8.0) followed by sample loading onto the membrane collecting the flow through in 5 mL fractions. After a 20 CV wash step using the no salt buffer, a linear gradient from 0 to 1 M NaCl over 20 CV was performed collecting the eluate in 1 mL fractions. All steps were performed at 5 mL/min.

### Transmission electron microscopy

Samples were diluted to approximately 20 μg/mL and deposited on carbon type-B, 200-mesh copper grids (Ted Pella Inc.). Grids had previously been treated with an easiGlow glow discharge unit (PELCO). After a 5 min incubation at room temperature, the grids were washed with distilled water and stained with 2% aqueous uranyl acetate (BDH Chemicals) for 7 min. Excess uranyl acetate was absorbed with filter paper (Whatman), and the grids were left to dry before visualization under a Tecnai G2 20 TEM (FEI).

## Supplementary Information

**Additional file 1: Figure S1.** SDS-PAGE analysis of the retentate and permeate fractions produced during tangential flow filtration. A small quantity of bacterial proteins passes through the membrane during the early stages of TFF (less than one volume). By two volumes, the system has nearly reached equilibrium. The protein content in the retentate is virtually unchanged throughout the whole procedure. **Figure S2.** Lysate samples used for multimodal chromatography purification of gamma-prefoldin. Cell lysate was prepared by lysis of *E. coli* cells in a low salt buffer (pH 8), followed by DNase I treatment and clarification by centrifugation (rightmost lane). Subsequently, the lysate was subjected to TFF (leftmost lane). The TFF-processed sample was then aliquoted and dialyzed into other buffers that vary in salt and pH conditions. None of these procedures significantly changed the total protein content, nor the amount of gamma-prefoldin (arrow), in each sample. LS: low salt; NS: no salt; HS: high salt; AS: ammonium sulphate. See Table 1 for more details on each buffer condition. **Figure S3.** Comparison of the early MMC elution fractions between samples that had been processed or not processed previously by tangential flow filtration. The first eleven fractions (corresponding to 11 column volumes) from a MMC purification of recombinant gamma-prefoldin from bacterial lysate were analyzed by SDS-PAGE. TFF pre-processing (gel on the left) of the lysate resulted in higher purity and delayed saturation of the resin (indicated by the appearance of protein contaminants) compared to the same lysate without TFF pre-processing (gel on the right). **Figure S4.** Comparison of initial fractions from MMC purification of recombinant gamma-prefoldin performed with different buffered solvents. The comparison of the third elution fraction from each MMC run (also shown in Fig. 2d) shows that MMC performs comparably in all solvents at the early stages of MMC, that ammonium sulfate caused some precipitation of gamma-prefoldin, and that omitting TFF pre-processing of the lysate in lesser purity of gamma-prefoldin. A comparison of a later fraction (the seventh elution fraction) reveals slight differences in performance between solvents, with some fractions exhibiting the first signs of resin saturation (greater amounts of bacterial protein contamination). **Figure S5.** Extended UV chromatograms of MMC purifications of recombinant gamma-prefoldin with varying buffer conditions. **(a)** The binding capacity of MMC for gamma-prefoldin is comparable in a range of buffered solvents during the early stages of chromatography. The onset of resin saturation is approximately 5 column volumes for all solvents (see Fig. 2c for a detailed view). Omitting the TFF pre-processing (dashed line) results in an earlier and steeper onset of resin saturation. **(b)** A zoomed in version of the same dataset, revealing small differences in the exact volume of the onset of resin saturation. CV: column volume. **Figure S6.** Transmission electron microscopy of gamma-prefoldin shows differences in the morphology of non-functionalized and functionalized protein filaments. **(a)** Gamma-prefoldin-SpyTag (γPFD-SpyT) forms filaments with similar morphology to wild type gamma-prefoldin (see Fig. 1f), although with shorter average filament lengths. **(b)** γPFD-SpyT functionalized with mCerulean3-SpyCatcher (mC3-SpyC) and mVenus-SpyCatcher (mV-SpyC) shows a different morphology, resembling concatenated “beads”. **Figure S6.** Transmission electron microscopy of *Q. thermotolerans* encapsulin. Encapsulin was co-expressed with mNeon fused to a targeting peptide that interacts with the inner face of the encapsulin nanocompartment, effectively packaging mNeon during encapsulin self-assembly. Encapsulin nanocompartments were purified by MMC followed by TX-114 phase partitioning, then imaged by TEM. TEM images showed mostly fully assembled nanocompartments. The presence of partially assembled or collapsed nanocompartments may be due to the dehydration of samples during TEM sample preparation. **Figure S7.** Purification of γPFD anion exchange chromatography. SDS-PAGE showing the purification of γPFD using Nano Q membrane adsorbers L: load; FT: flow-through; E: eluate.

## Abbreviations

γPFD: Gamma-prefoldin
AEX: Anion exchange
CC700: Capto Core 700
CFP: Cyan fluorescent protein
FP: Fluorescent protein
MMC: Multimodal chromatography
PPI: Protein–protein interaction
SpyC: SpyCatcher
SpyT: SpyTag
TFF: Tangential flow filtration
TX-114: Triton X-114
YFP: Yellow fluorescent protein
